# Supplementary material for: Identifying interventions to reduce peripartum haemorrhage associated with caesarean delivery in Africa: A Delphi consensus study
Source: PLOS Glob Public Health. 2022 Aug 31;2(8):e0000455. doi: 10.1371/journal.pgph.0000455 (PMC10021587; doi:10.1371/journal.pgph.0000455)
Supplement: S1 Text — (DOCX) [file pgph.0000455.s001.docx]

# Supporting information 1 (S1 Text). Members of the APORG Caesarean Delivery Haemorrhage Group

**Writing Committee:**

Elliott H Taylor,^1^ Salome Maswime,^2^ David Bishop,^3^ Fred Bulamba,^4^ Rowan Duys,^5^ Robert Dyer,^6^ Sue Fawcus,^7^ Thomas O Konney,^8^ Milton W Musaba,^9^ Jolene Moore,^10^ Dolly M Munlemvo,^11^ Akinyinka Omigbodun,^12^ Kokila Lakhoo,^13^ Bruce M Biccard,^14^ on behalf of the APORG Caesarean Delivery Haemorrhage Group.

1. Dr Elliott H Taylor, MBBS, Global Surgery Division, Department of Surgery, University of Cape Town, South Africa and Oxford University Global Surgery Group, Oxford, United Kingdom
2. Associate Professor Salome Maswime, PhD, Global Surgery Division, Department of Surgery, University of Cape Town, South Africa
3. Honorary Associate Professor David G Bishop, PhD, Department of Anaesthetics, University of KwaZulu-Natal, Durban, South Africa
4. Dr Fred Bulamba, Mmed, Department of Anesthesia and Critical Care, Faculty of Health Sciences Busitema University, Uganda
5. Dr Rowan Duys, FCA (SA), Department of Anaesthesia and Perioperative Medicine and Global Surgery Division, University of Cape Town, South Africa
6. Professor Robert Dyer, PhD, Department of Anaesthesia and Perioperative Medicine, University of Cape Town, South Africa
7. Professor Sue Fawcus, FRCOG (UK), Emeritus Professor and Senior Research Scholar, Department of Obstetrics and Gynaecology, University of Cape Town, Cape Town, South Africa
8. Dr Thomas O Konney, FWACS, Komfo Anokye Teaching Hospital Department of Obstetrics and Gynaecology, Kumasi, Ghana
9. Dr Milton W Musaba, PhD, Busitema University Faculty of Health Sciences and Mbale Regional Referral Hospital, Uganda
10. Dr Jolene Moore, MBChB, School of Medicine, Medical Sciences and Nutrition, University of Aberdeen, Aberdeen, United Kingdom
11. Dr Dolly M Munlemvo, MD, University of Kinshasa, Kinshasa, Democratic Republic of the Congo and Nationwide Children's Hospital, Columbus, Ohio, United States
12. Professor Akinyinka O Omigbodun, FWACS, Department of Obstetrics and Gynaecology, College of Medicine, University of Ibadan, Nigeria
13. Professor Kokila Lakhoo, PhD, Nuffield Department of Surgical Sciences, University of Oxford, Oxford, United Kingdom
14. Professor Bruce M Biccard, PhD, Professor and 2nd Chair, Department of Anaesthesia and Perioperative Medicine, Groote Schuur Hospital, Faculty of Health Sciences, University of Cape Town, South Africa

**Operations Committee (alphabetical):**

Bruce Biccard *(Groote Schuur Hospital, Faculty of Health Sciences, University of Cape Town, South Africa)*, Freddy F Kabambi *(Nelson Mandela Academic Hospital, Walter Sisulu University, South Africa)*, Kokila Lakhoo *(Nuffield Department of Surgical Sciences, University of Oxford, Oxford, United Kingdom)*, Salome Maswime *(Global Surgery Division, Department of Surgery, University of Cape Town, South Africa)*, Dawid van Straaten *(Safe Surgery South Africa, Gauteng, South Africa)*, Elliott H Taylor *(Global Surgery Division, Department of Surgery, University of Cape Town, South Africa and Oxford University Global Surgery Group, Oxford, United Kingdom).*

**Delphi Experts (alphabetical):**

Angola: Leyandis Cobas *(Clínica Sagrada Esperança, Luanda).*

Botswana: Tadele Melese Benti *(Princes Marina Hospital, University Of Botswana),* Mamo Woldu Kassa *(University Of Botswana),* Gaone Kediegile *(University Of Botswana)*.

Burkina Faso: Ouedraogo Nazinigouba *(Chu Yalgado Ouédraogo).*

Burundi: Ndayisaba Carter *(Kira Hospital)*, Harerimana Salvator *(University Teaching Hospital).*

Democratic Republic Of The Congo: Mubeya Franck *(General Hospital Of Kinshasa)*, Ted B Likongo *(El Rapha Clinic)*, Mukenga Mamba Martin *(University Hospital Of Kinshasa)*, Dolly M. Munlemvo (*University of Kinshasa, Kinshasa and Nationwide Children's hospital, Columbus, Ohio).*

Egypt: Maher Fawzy *(Cairo University Hospitals)*, Ashraf Nabhan *(Department of Obstetrics and Gynecology, Ain Shams University).*

Ethiopia: Jolene Moore *(Debre Birhan Comprehensive Specialized Hospital)*, Melat Sebsibie *(Saint Peter Specialized Hospital),* Rediet Shimeles Workneh *(Ghandi Memorial Hospital).*

Ghana: Thomas O Konney *(Komfo Anokye Teaching Hospital Department of Obstetrics and Gynaecology, Kumasi).*

Kenya: Alex N Bosire *(University of Nairobi),* Timothy M Mwiti *(Department of Anaesthesia, University of Nairobi)*.

Libya: Rabie Salem Alfetouri *(National Cancer Institute).*

Madagascar: Rajaonarison Tahina Joëlle *(Centre Hospitalier De Gynecologie Obtetrique De Befelatanana, Tananarive),* Razafindrainibe Tanjonirina *(Centre Hospitalier De Gynecologie Obtetrique De Befelatanana, Tananarive).*

Malawi: Delia C Mabedi *(Zomba Central Hospital)*, Priscilla Mvula-Mtila *(Kamuzu University of Health Sciences, Department of Obstetrics and Gynaecology).*

Mali: Boubacar Diallo *(University Hospital Center of Point G, Department of Anesthesia intensive care medicine and Emergencies, Bamako),* Moustapha Issa Mangané *(University Hospital Center of Gabriel Touré, Department of Anesthesia intensive care medicine and Emergencies, Bamako).*

Mauritius: Vakil Leellodharry *(Ministry of Health and Quality of Life).*

Mozambique: Emila Jeque *(Hospital Central De Maputo*), Magda Ribeiro *(Hospital Central De Maputo).*

Niger: Nayama Madi *(Maternité Issaka Gazobi),* Idrissa Rekia *(Centre Hospitalier Régional De Niamey).*

Nigeria: Abiodun Aboyeji *(University Of Ilorin Teaching Hospital),* Akinyinka Omigbodun *(University Of Ibadan)*.

Rwanda: Magnifique Irakoze *(University of Rwanda),* David Ntirushwa *(University Teaching Hospital of Kigali (CHUK) / University of Rwanda , Department of Obstetrics and Gynecology),* Mvukiyehe Jean Paul *(University Teaching Hospital of Butare (CHUB)/ University of Rwanda, Department of Anesthesia and Critical Care)*, Eugene Tuyishime *(Anesthesia Critical Care and Emergency Medicine department, University of Rwanda, Kigali).*

Sierra Leone: Valerie John-Cole *(PCM Hospital)*, Omobowale G. Olopade *(PCM Hospital)*, Ahmadu Sesay *(PCM Hospital)*.

South Africa: Motselisi Mbeki *(Kalafong Provincial Tertiary Hospital),* Felicia Molokoane *(Kalafong Provincial Tertiary Hospital).*

Tanzania: Gerald‎ Cubwa *(Uwata Hospital),* Gloria Kinasa *(Rabininsia Memorial Hospital)*, Letisia Frostan Komba *(Mbeya Zonal Referral Hospital)*, Amos Mazuka *(Mbeya Zonal Referral Hospital*).

The Gambia: Sulayman Jallow *(Edward Francis Small Teaching Hospital),* Abdoulie Keita *(Edward Francis Small Teaching Hospital)*, Musa Marena *(Edward Francis Small Teaching Hospital and Faculty of Medicine & Allied Health Science, University Of The Gambia),* Anna Njie *(Edward Francis Small Teaching Hospital)*, Masirending Njie *(Edward Francis Small Teaching Hospital).*

Uganda: Fred Bulamba *(Department of Anesthesia and Critical Care, Faculty of Health Sciences Busitema University),* Milton W Musaba *(Department of Obstetrics and Gynaecology, Busitema University/ Mbale Regional Referral Hospital).*

Zambia: Ushmaben Patel-Mujajati *(Medland Hospital),* Angel Phiri *(University Teaching Hospitals - Women and Newborn Hospital)*
