# Supplementary material for: Identifying interventions to reduce peripartum haemorrhage associated with caesarean delivery in Africa: A Delphi consensus study
Source: PLOS Glob Public Health. 2022 Aug 31;2(8):e0000455. doi: 10.1371/journal.pgph.0000455 (PMC10021587; doi:10.1371/journal.pgph.0000455)
Supplement: S1 Data — (DOCX) [file pgph.0000455.s002.docx]

# Supporting Information (S1 Data). Summarised Delphi scores

| ** upgraded to the final list of recommended interventions in round four.* | Round Two Scores | | Round Three Scores | |
| --- | --- | --- | --- | --- |
|  | Effectiveness, median  (IQR) | Feasibility, median (IQR) | Effectiveness, median  (IQR) | Feasibility, median  (IQR) |
| **Direct Interventions - Antenatal care and Assessment** | | | | |
| Routine pre-operative risk assessment and risk stratification for peripartum haemorrhage | 8 (7-9) | 7 (6-8) | 8 (7-9) | 7 (7-8) |
| Routine screening for anaemia and patient blood management programme | 7 (7-8.5) | 7 (5-8) | 8 (7-8) | 7 (6-8) |
| Routine antenatal assessment by the anaesthesia provider for all women for planned caesarean delivery. | 8 (7-9) | 8 (5-8) | 8( 7-9) | 7 (6-8) |
| Maternal education on the early self-recognition of signs and symptoms of peri-partum haemorrhage. | 7 (6-8) | 7 (6-8) | 7 (6-8) | 7 (6-8) |
| **Direct Interventions - Peri-operative Care** | | | | |
| Routine use of a Surgical Safety Checklist modified for caesarean delivery use. | 8 (6.25-9) | 7 (6-8.75) | 8 (7-9) | 8 (7-8) |
| Implementation of a standardised peri-partum haemorrhage protocol. | 9 (7.75-9) | 9 (7-9) | 9 (8-9) | 8 (7-9) |
| Intrapartum monitoring: routine, close monitoring of mother and foetus during intrapartum period. | 8 (7-9) | 7 (6-8) | 8 (7-8.25) | 7 (6-8) |
| Early and increased surveillance of vital signs in the postpartum period, possibly in a dedicated environment. | 8 (7-9) | 7 (6-8) | 8 (7-9) | 7 (6-8) |
| *Routine use of modified early obstetric warning score in the postoperative period, or routine active monitoring for haemorrhage e.g. shock index. | 7 (7-9) | 6 (6-8) | 7 (7-8) | 6 (6-8) |
| Early involvement of anaesthesia providers in the management of patients at risk. | 8 (7-9) | 7 (6-9) | 8 (7-8) | 8 (7-8) |
| Availability of a peri-partum haemorrhage ‘package’ containing all items required to manage post-partum haemorrhage. | 8 (7-9) | 7 (6-7) | 8 (7-9) | 7 (6-7) |
| Availability of emergency blood products. | 9 (8-9) | 7 (5.75-8) | 9 (8-9) | 7 (5-8) |
| Availability of first line uterotonic agents. | 9 (8-9) | 8 (7-9) | 9 (8-9) | 8 (8-9) |
| Availability of alternative/second line uterotonic agents. | 8 (7-9) | 7 (6-9) | 8 (8-9) | 7 (7-8) |
| Availability of tranexamic acid. | 8 (7-9) | 7 (6-9) | 8 (7-8) | 8 (6-8) |
| Ability to perform active management of the third stage of labour. | 9 (8-9) | 9 (7.75-9) | 9 (8-9) | 9 (8-9) |
| Ability to perform uterine massage and bimanual compression. | 8 (7-9) | 8 (7-9) | 8 (7-9) | 8 (7-8) |
| Ability to perform hysterectomy and uterine artery ligation. | 9 (8-9) | 7 (6-9) | 8 (8-9) | 7 (6-8) |
| *Ability to perform B-lynch and compression sutures. | 8 (7-9) | 7 (5-8) | 8 (7-8) | 6 (5-7) |
| *Ability to perform balloon tamponade. | 8 (6-8.25) | 6.5 (5-8) | 7 (6-8.25) | 6 (5-8) |
| **Indirect Interventions - Community Based** | | | | |
| Community information, education and communication regarding reproductive health (e.g. knowledge on risks, the early recognition of danger signs in pregnancy, and the advantages of seeking healthcare at an early stage). | 7 (6-8) | 6 (5-8) | 7 (7-8) | 7 (6-7) |
| Availability of contraception and family planning. | 7 (6-9) | 6 (4-8) | 7.5 (7-9) | 7 (6-8) |
| **Indirect Interventions - Health System Strengthening** | | | | |
| Ensuring a minimum training standard for healthcare providers. | 8.5 (8-9) | 8 (6-8.25) | 8 (8-9) | 8 (7-8) |
| Advocating to key stakeholders about the importance of reducing maternal morbidity and mortality related to peripartum haemorrhage. | 8.5 (7.75-9) | 7 (6-9) | 8 (7-9) | 7 (6.75-8) |
| Establishing smooth and timely referral patterns between levels of care. | 8 (7-9) | 7 (6-8) | 8 (7.75-9) | 7 (6-8) |
| Reducing delays in receiving care once at the healthcare facility. | 8 (8-9) | 7 (6-8) | 8 (8-9) | 7 (6-8) |
| Multidisciplinary simulation and team training. | 8.5 (8-9) | 7 (6-8) | 8 (8-9) | 7 (6-7) |
| *Available resources to perform caesarean delivery 24 hours a day. | 9 (8-9) | 7 (6-8.25) | 9 (8-9) | 6 (5.75-7) |
| **Interventions not included in the final list of recommended intervention** | | | | |
| Availability of a specialist obstetrician and anaesthetist. | 9 (8-9) | 6 (5-7) | 9 (8-9) | 6 (4-7) |
| Early, comprehensive antenatal consultation with regular follow up. | 8 (5.5-9) | 7 (6-8) | 8 (6.75-8) | 6.5 (6-8) |
| Providing mentorship and support networks to non-physician surgery providers and non-physician anaesthesia providers. | 8 (6-9) | 7 (6-8) | 8 (6-8.25) | 6 (5.75-7) |
| Creation of multidisciplinary peri-partum haemorrhage response team with a rapid alert and call network. | 8 (7-9) | 6 (5-7) | 8 (7-9) | 6 (5-7) |
| Ensuring repeat caesarean delivery are only performed by experienced providers. | 8 (6.75-9) | 6 (4.75-7) | 8 (7-8) | 6 (5-7) |
| Accurate and quantitative measurement of peripartum blood loss. | 7 (5-8) | 6 (5-7) | 7 (6-8) | 6 (5-7) |
| Routine ultrasound localisation of the placenta, prior to labour. | 7 (6-8) | 5 (4-7) | 7 (6-8) | 5 (4-7) |
| Availability of anti-shock garments. | 7 (5-8) | 5.5 (4-7) | 7 (6-7) | 5 (3-6) |
| A routine pre-operative coagulation assessment. | 6 (4-7.5) | 5 (3-6) | 6 (4-7) | 5 (3-6) |
| Intraoperative blood salvage. | 6.5 (5-8.25) | 4 (2.75-6) | 6 (5.75-8) | 4 (3-5) |
| Ability to perform arterial embolization. | 7 (4-8) | 3 (1.75-4) | 7 (5-8) | 3 (2-4) |
