# Supplementary material for: Identifying interventions to reduce peripartum haemorrhage associated with caesarean delivery in Africa: A Delphi consensus study
Source: PLOS Glob Public Health. 2022 Aug 31;2(8):e0000455. doi: 10.1371/journal.pgph.0000455 (PMC10021587; doi:10.1371/journal.pgph.0000455)
Supplement: Attachment — Submitted filename: Response to Reviewers.doc [file pgph.0000455.s003.doc]

 30 November 2026

#	**UNIVERSITY OF CAPE TOWN**

**Professorial Staff:**

JLC Swanevelder MB ChB, MMed (Anes) (U Stell), FCA (SA), FRCA (Hon)

Head of Department

Email: justiaan.swanevelder@uct.ac.za

BM Biccard MBChB (UCT), FCA (SA), FFARCSI, MMed (UKZN), PhD

2^nd^ Chair of Anaesthesia
Email: [bruce.biccard@uct.ac.za](mailto:bruce.biccard@uct.ac.za)
Administrative Officer: C Wyngaard, Email: cheryl.wyngaard@uct.ac.za

## **Department of Anaesthesia and Perioperative Medicine**

Faculty of Health Science,
Anzio Road, Observatory
Western Cape, South Africa 7925
Telephone: (021) 406-6143
Fax No. (021) 406-6589
GSH departmental line: (021) 404-5004

Professor Dylan Bould

Academic Editor, *PLOS Global Public Health*

**Revision: Manuscript reference number: PGPH-D-22-00210 – Identifying interventions to reduce peripartum haemorrhage associated with caesarean delivery in Africa: a Delphi consensus study**

Dear Professor Dylan Bould,

Thank you for the opportunity to submit a revision of our paper for publication in *PLOS Global Public Health*. We have itemised the queries with a written description of our responses and amendments.

Should this paper be accepted for publication, we wish to publish the paper under PubMed citable collaborative authorship – ‘African Perioperative Research Group (APORG) - Caesarean Delivery Haemorrhage Group’. PubMed citable collaborators are listed in Supporting information 1 (S1).

We believe this revision is significantly improved compared to the original submission, thanks to the reviewers’ insightful comments and queries. We would again like to express our gratitude for the constructive comments and opportunity to revise our paper.

Regards

Bruce Biccard, on behalf of the African Perioperative Research Group (APORG) - Caesarean Delivery Haemorrhage Group

**Reviewer 1:**

**Comment/query 1:**

Thank you for the opportunity to review this manuscript of a Delphi consensus to establish a context-specific list of feasible and effective strategies to reduce mortality from peripartum hemorrhage with caesarean section in Africa. This research is timely and necessary within the Sustainable Development Goals framework and the African Perioperative Research Group priorities. The variety of interventions considered, ranging from the provider to systems level, was highly pragmatic and comprehensive. It was informative to read about specific examples of recommendations that have been successfully implemented and sustained in various African countries. The paper also serves as a robust foundation for future work focused on operationalizing these interventions.

Response 1:

We would like to thank the reviewer for their comments and interest in our research.

**Comment/query 2:**

Background: The authors state that it is unclear whether current interventions to reduce maternal mortality are implementable in low resource settings. The authors could provide more information about pre-existing guidelines with focus on who developed them and the intended target population, to strengthen the argument that they may not be applicable to their specific context in Africa.

Response 2:

We thank the reviewer for highlighting this. The book chapter to which we refer (8) provides a comprehensive list of interventions rather than guidelines. The purpose of our paper is to outline interventions which African researchers and clinicians consider to be both effective and feasible in the African context. We do discuss the applicability of suggested guidelines from high-income countries in the Discussion, and would prefer not to elaborate further on currently used guidelines across Africa in the introduction. We did not conduct analysis on which guidelines are currently referred to across Africa and thus feel that commenting on this aspect may be speculative.

**Comment/query 3:**

Methods: The non-random sampling technique should be further described. Why did the authors decide to only approach leaders from the African Surgical Outcomes Study and/or African Surgical Outcomes-2 Trial? Were there considerations to reach out to other national leaders.

Response 3:

Thank you for this query. The African Perioperative Research Group (APORG) – is a network of clinicians and researchers in hospitals across Africa. The major trials undertaken by APORG thus far are (i) the African Surgical Outcomes Study (PMID: 29306587 + 30879511) and (ii) the African Surgical Outcomes-2 Trial (PMID: 34418380). In continental research priority setting, APORG highlighted the *‘early identification and management of mothers at risk from peripartum haemorrhage in the peri-operative period’* (PMID: 31903576). APORG now includes over 1400 clinician investigators. We therefore considered it appropriate to continue to work with collaborators within the APORG network. National leaders from the APORG network were asked to recommend experts from within their country. National leaders were not involved in this research unless they had direct involvement in obstetric care or obstetric anaesthesia within their country. Other collaborative groups were not approached for involvement in this study. We have amended the following sentence under ‘Participant selection’ to give clarity:

*“To identify African obstetricians and anaesthetists considered leaders in the field of obstetric care and obstetric anaesthesia, we asked National leaders from previous APORG studies - the African Surgical Outcomes Study^5^ and/or the African Surgical OutcomeS-2 Trial^10^ to identify and invite experts from within their respective countries. We encouraged the participation of two obstetric and anaesthesia providers from each country.”*

**Comment/query 4:**

Methods: During round two of the consensus, were participants given the list of interventions with or without the justifications and references? How many participants were involved in round 4? Did final consensus during round 4 require agreement from all present participants? A definition of consensus could be included. The wording of the 9-point Likert scale could also be included for methodological clarity.

Response 4:

Thank you for highlighting this. In round one participants proposed key interventions for consideration, and were not asked to provide justification/references at this stage. In round two participants were given the list of amalgamated and grouped interventions from round one and *“were encouraged to provide further justifications or references to support their scoring”*. Therefore, round three was the first opportunity for participants to view justifications/references alongside the respective interventions and their summarised scores (median and interquartile range). We have amended text in the Results section for clarification:

*“In round three, 40 participants re-scored the interventions, while considering the group scores (median and IQR) (supporting information 2) and anonymised justifications/references from round two.”*

The Round 4 live discussion involved 30 participants – we have amended the Results section to include this:

*“In round four, 30 participants took part in the online discussion. Four ‘potential interventions’ were upgraded to the final list of recommended interventions.”*

No formal definition of consensus was used for Round 4, though participants were given opportunity to support/reject upgrading any of the fourteen ‘potential interventions’ considered to have moderate effectiveness (median 4-6) and/or moderate feasibility (median 4-6). There were no disagreements within the group regarding decisions made in the round four meeting.

The definition of the Likert scale is provided in the manuscript as follows:

*“A score of 1-3 was considered low effectiveness/feasibility (not recommended); 4-6 was considered moderate effectiveness/feasibility (possibly recommended); and 7-9 was considered high effectiveness/feasibility (recommended).”*

**Comment/query 5:**

Results: The authors should consider listing the interventions that were excluded from the final list as this can provide equally informative data for readers. Furthermore, it would be interesting to receive the associated explanations as to their lack of feasibility and/or effectiveness.

Response 5:

Thank you for this comment. The excluded interventions are listed in ‘Supporting information 2 (S2). Summarised Delphi scores’. Though participants were *“encouraged to provide further justifications or references to support their scoring”*, it was not mandatory to do so. We therefore do not have complete data on this aspect; and there was no planned qualitative analysis of this data. To our knowledge such data is not routinely presented in Delphi studies, but future studies should consider this aspect.

**Comment/query 6:**

Results: The authors can consider including the following source about the development of postpartum hemorrhage bundles, which supports several perioperative interventions: Althabe F, Therrien MNS, Pingray V, Hermida J, Gülmezoglu AM, Armbruster D, et al. Postpartum hemorrhage care bundles to improve adherence to guidelines: A WHO technical consultation. Int J Gynecol Obstet. 2020;148(3):290–9.

Response 6:

Thank you for this suggestion. We have included the source in the following discussion when referring to haemorrhage packages/bundles:

*“Obstetric haemorrhage packages/bundles exist^24,25^ though the development and assessment of packages/bundles contextualised for caesarean delivery in low-resource settings is needed.”*

**Comment/query 7:**

Results: More background information regarding the current situation of availability of 24 h emergency caesarean section services should be included.

Response 7:

Thank you for this comment. Though we are unable to identify quantitative evidence on the current situation regarding access to 24-hour caesarean section across Africa, we have added further detail to the discussion regarding the low caesarean delivery rate across Africa (given that caesarean delivery rate acts as a surrogate for access to caesarean delivery), and how 24-hour access may improve this:

*“Emergency caesarean delivery may be required at any time of day. The ability to provide a safe 24-hour caesarean delivery service may reduce delays in receiving care and unsafe referrals between healthcare facilities. In addition, improving 24-hour access to caesarean delivery may contribute to increasing the caesarean delivery rate across Africa, to rates associated with a reduction in maternal mortality.^4,3^”*

**Comment/query 8:**

The logical flow on page 16, lines 319-325 is unclear and could be improved

Response 8:

Thank you for highlighting this. We have amended the text as follows and hope this has improved the logical flow:

*“A meta-analysis of maternal mortality after caesarean delivery found that women are more likely to die at teaching and tertiary hospitals.^29^ National audit data from the National Committee on Confidential Enquiries into Maternal Deaths in South Africa show that many caesarean deliveries were performed at district hospitals and when complications arose women were subsequently referred to higher levels of care (regional, tertiary and national hospitals), where they then died.^28^ These data emphasise the need to improve referral systems, to ensure timely referral and transfer of women at high risk of peripartum haemorrhage”*

**Reviewer 2:**

**Comment/query 9:**

A well written paper, and very valuable study on a very important clinical issue/challenge in Global Health.
Delphi study was appropriate to obtain expert consensus.
Perhaps more commentary on implementing policies to support the interventions outlined would enhance the discussion.

Response 9:

We thank the reviewer for their comments and interest in our research. We have added the following reference to the discussion which may be of use during intervention implementation:

*“Frameworks which measure the impact of such interventions within the wider healthcare system, may be of use.^31”^*

*31. Donabedian A. Evaluating the quality of medical care. Milbank Q. 2005;83(4):691–729.*

**Reviewer 3:**

**Comment/query 10:**

This is a well conducted and presented paper addressing a common cause of maternal mortality. Care bundles are commonly referred to and used for obstetric hemorrhage (see Joint Council) and this paper addresses the peri-operative care bundle, particularly with respect to haemorrhage. (The one by the Joint Council may be referenced, even though it is more US based.)

Response 10:

We thank the reviewer for their comments and interest in our research. Thank you for suggesting this reference, which we have included in the discussion when referring to packages/bundles:

*“Obstetric haemorrhage packages/bundles exist^24,25^ though the development and assessment of packages/bundles applicable for caesarean delivery in low-resource settings is needed.”*

**Comment/query 11:**

The paper correctly included upstream (public health and proximate areas) that can be part of the care bundle.

Response 11:

Thank you for highlighting this, we agree that focus on ‘upstream’ interventions is vital.

**Comment/query 12:**

All four rounds and the iterative process have been explained (Rounds 1,2, and 3 and finally the Round 4 discussion for remaining consensus). Noted that started with forty-four experts in round 1, which dropped to 41 and 40 in rounds 2 and 3 respectively. Did all forty-four rejoin in round 4?

Response 12:

The Round 4 live discussion involved 30 participants – we have amended the Results section to include this:

*“In round four, 30 participants took part in the online discussion. Four ‘potential interventions’ were upgraded to the final list of recommended interventions.”*

**Comment/query 13:**

Of the 14 potential interventions considered to have moderate effectiveness (median 4-6) and/or moderate feasibility (median 4-6), for consideration in the round four consensus meeting, four borderline interventions were upgraded to the final list of recommended interventions. The reader can see the remaining ten interventions that did not make it to the final list. This is very useful for different countries to weigh the merits and demerits of those that were not included. This author noted a few not included that are constantly addressed in his setting as issues of concern based on regular maternal mortality reviews.

Response 13:

Thank you for this comment; we agree that listing the excluded interventions is potentially very useful.

**Comment/query 14:**

Strength – included obstetricians and anesthetists. The affiliations of the experts are noted. Were the specialists from different types of hospitals? For example, large teaching hospitals down to district level hospitals? Might this have made a difference?

Response 14:

Thank you for highlighting this. This data is presented in the Results section, and described as a possible limitation in the Discussion section:

Results: *“34/44 (77%) of participants worked in a tertiary facility, 8/44 (18%) at a regional facility and 2/44 (5%) at a district/rural facility.”*

Discussion; Limitations: “*Since the Delphi study was conducted online, this study is biased towards receiving data from those with access to email and the internet, and the majority of input was received from participants working in tertiary healthcare settings. This may have resulted in the omission of critical insights from those working in district and rural healthcare facilities.*”

**Comment/query 15:**

The Limitations correctly mention that ‘no patients, midwives or public health specialists were involved in the Delphi process’. Agree that the involvement of these stakeholders may have widened the scope of recommendations. ‘Patient experience’ is becoming a useful tool to understand issues around quality of care. In the circumstance of peri-operative care, the views of theatre nurses and particularly recovery-ward nurses, if included, could have added more information. Nevertheless, a few ‘direct items’ like those related to early and increased surveillance and consideration of the monitoring for the shock index are what they may have brought up.
Thank you

Response 15:

Thank you, we agree that future studies should include various other stakeholders.

**Journal Requirements:**

**Journal Requirement 1:**

Please provide separate figure files in .tif or .eps format only.  Please ensure that all files are under our size limit of 20MB.

Response 1:

We have converted and uploaded all figures in TIFF format.

**Journal Requirement 2:**

Please provide us with a direct link to the base layer of the map used in Fig 2 and ensure this location is also included in the figure legend.

Response 2:

Fig 2 was created at <https://www.mapchart.net/>. Created maps are licensed under a [Creative Commons Attribution-ShareAlike 4.0 International License](http://creativecommons.org/licenses/by-sa/4.0/)). A link to mapchart.net has been added to the figure legend.
